# Clinical Implications of Kinesiotaping for Forearm Muscle Function: Acute Effects on Grip Strength, Pain Sensitivity, and Muscle Oxygenation in Healthy Active Adults: A Randomized Controlled Trial

**DOI:** 10.3390/healthcare13243211

**Published:** 2025-12-08

**Authors:** José Ángel del-Blanco-Muñiz, Arturo Ladriñán-Maestro, Guillermo Vergara-Gatica, Cristóbal Orellana-García, Guillermo García-Pérez-de-Sevilla, Daniel Martín-Vera, Alberto Sánchez-Sierra

**Affiliations:** 1 Department of Rehabilitation, Faculty of Medicine, Health and Sports, Universidad Europea de Madrid, 28670 Madrid, Spain; joseangel.delblanco@universidadeuropea.es (J.Á.d.-B.-M.); guillermo.garcia@universidadeuropea.es (G.G.-P.-d.-S.); daniel.martin2@universidadeuropea.es (D.M.-V.); 2 Grupo de Investigación en Ejercicio Terapéutico y Rehabilitación Funcional, Faculty of Medicine, Health and Sports, Department of Rehabilitation, Universidad Europea de Madrid, 28670 Madrid, Spain; 3 Faculty of Physiotherapy and Nursing of Toledo, Universidad de Castilla-La Mancha, 45071 Toledo, Spain; alberto.sanchez@uclm.es; 4 Pain, Mental Health, Exercise and Technology Research Group (PAIN + MET), Faculty of Physical Therapy and Nursing, Universidad de Castilla-La Mancha, 45071 Toledo, Spain; 5 Department of Physiotherapy, Faculty of Medicine, Health and Sports, Universidad Europea de Madrid, 28670 Madrid, Spain; guillermovergara.g@gmail.com (G.V.-G.); cris.orellanag1@gmail.com (C.O.-G.); 6 Grupo de Investigación en Fisioterapia Toledo (GIFTO), Facultad de Fisioterapia y Enfermería, Universidad de Castilla-La Mancha, 45071 Toledo, Spain; 7 Grupo de Investigación en Fisioterapia Toledo (GIFTO), Instituto de Investigación Sanitaria de Castilla-La Mancha, 45071 Toledo, Spain

**Keywords:** grip strength, kinesiotaping, muscle oxygen saturation, NIRS, pain

## Abstract

**Highlights:**

**What are the main findings?**

- Only proximal-to-distal kinesiology taping produced a significant increase in muscle oxygenation (SmO_2_) after fatiguing exercise.
- No significant intergroup differences were observed in grip strength, pressure pain threshold, or total hemoglobin.

**What are the implications of the main findings?**

- The KT direction may transiently enhance local muscle oxygenation, suggesting a potential circulatory effect.
- Further studies are needed to determine the clinical or performance relevance of these acute physiological responses.

**Abstract:**

**Background/Objective:** Kinesiotaping (KT) is widely used in sports and rehabilitation; however, evidence regarding its acute physiological effects on strength, pain sensitivity, and muscle oxygenation remains inconsistent. This study aimed to examine the acute effects of forearm KT on maximal grip strength, pressure pain threshold (PPT), and muscle oxygenation in healthy, physically active adults who performed a fatiguing exercise protocol. **Methods:** A randomized controlled trial was conducted with 28 participants (56 forearms), each randomly assigned to one of four conditions: KT applied proximal-to-distal, KT applied distal-to-proximal, placebo taping (no tension), or no taping (control). All assessments were performed within a single session, before and after a standardized forearm fatigue protocol. The outcomes included maximal and average grip strength (digital dynamometer), PPT (digital algometer), muscle oxygen saturation (SmO_2_) and total hemoglobin (THb) measured using near-infrared spectroscopy (NIRS). Data were analyzed using two-way ANOVA (time × group) with Bonferroni-adjusted post hoc tests. **Results:** All groups showed significant within-group reductions in grip strength after the fatigue protocol (Δ −2.8 to −7.9 kg; all *p* ≤ 0.01), confirming the effectiveness of fatigue induction. Between-group analysis revealed a significant effect only for SmO_2_ (*p* < 0.001; η^2^p = 0.317), with the proximal-to-distal KT group showing the largest post-fatigue increase (Δ +22.4; *p* < 0.001; Cohen’s d = 2.99) in SmO_2_. However, a comparable increase in SmO_2_ was also observed in the control group, suggesting a possible nonspecific reperfusion or oxygenation recovery effect. No between-group differences were observed in THb (*p* = 0.061), maximal grip strength (*p* = 0.092), average grip strength (*p* = 0.465), or PPT (*p* = 0.431). **Conclusions:** In healthy, physically active adults, forearm kinesiotaping did not produce significant acute effects on grip strength, pain threshold, or total hemoglobin levels following fatigue. Although a transient increase in SmO_2_ was observed with proximal-to-distal taping, this change likely reflects a non-specific post-fatigue reperfusion response rather than a direct enhancement of perfusion. These findings support a physiological, rather than clinical, interpretation of KT’s effects. Future studies should include clinical or athletic populations and explore whether repeated applications produce cumulative adaptations in muscle oxygenation and recovery processes.

## 1. Introduction

Kinesiotaping (KT) was developed by Dr. Kenzo Kase in Japan in the 1970s with the goal of creating an elastic therapeutic tape capable of supporting muscles and joints without limiting the range of motion. Beyond mechanical support, KT was designed to influence the somatosensory and lymphatic systems, potentially improving circulation, reducing pain, and facilitating tissue recovery. Over time, it has gained widespread use in sports and rehabilitation for its proposed benefits, such as enhancing proprioception, reducing muscle fatigue, improving neuromuscular activation, and promoting lymphatic drainage, although scientific evidence for these effects remains inconsistent and often comparable to placebo or standard care interventions [1,2].

KT utilizes an elastic, water-resistant cotton tape that can stretch for up to 60% of its original length. It allows full joint range of motion and can remain on the skin for three to five days without losing its effectiveness [3,4]. This technique has been promoted for its benefits in sports and functional rehabilitation, including improved proprioception, reduced muscle fatigue, pain reduction, increased neuromuscular activation, and lymphatic drainage [4,5]. However, the available scientific evidence is heterogeneous and often fails to demonstrate effects that are superior to those of a placebo or other standard interventions for musculoskeletal injuries [5,6].

Some studies have reported positive results in functional outcomes such as pain. For instance, a meta-analysis by Zhong et al., 2020 found that KT was effective in reducing pain, improving grip strength, and increasing functionality in patients with lateral elbow epicondylitis [7]. In contrast, other studies contradict these findings. Drapeza et al., 2022 concluded that KT had no significant effects on parameters such as wrist pain, grip strength, pinch strength, or overall upper limb function in patients with De Quervain’s disease, increasing uncertainty regarding its effectiveness in forearm muscle pathologies [8].

The literature presents inconsistent results regarding KT’s impact on grip strength in individuals with forearm musculoskeletal disorders. While some studies support its ability to increase strength, others have found no significant differences [6,9]. Importantly, specific evidence on the acute effects of KT on maximal grip strength in healthy, physically active individuals is very limited, representing a significant gap in current knowledge about its use for immediate performance enhancement or prevention [10].

Furthermore, scientific knowledge regarding the effect of KT on blood flow or deep muscle oxygenation is even more limited. Near-infrared spectroscopy (NIRS) is a promising technology for the objective and noninvasively assessment of changes in tissue oxygenation by measuring muscle oxygen saturation (SmO_2_) and total hemoglobin (THb) [11]. To date, only one study has used NIRS to investigate the acute effects of KT on muscle tissue injury. Carvalho et al., 2024 evaluated the effects of KT on the calf of patients with chronic venous insufficiency and found no relevant improvements in muscle oxygenation, leaving the discussion about its real physiological utility open [12].

Given the scarcity and inconsistency of available studies, there is a need for controlled research to clarify whether KT can have a real and measurable physiological impact, particularly on objective variables such as strength, pain, and tissue oxygenation. This study aimed to determine the acute effects of kinesiotaping direction on forearm function and local muscle oxygenation in healthy active adults as a first step toward informing clinical assessment and rehabilitation strategies in athletes and individuals with forearm overuse syndromes. We hypothesized that the direction of kinesiotaping application would influence the acute physiological and functional responses of the forearm muscles. Specifically, proximal-to-distal taping was expected to increase muscle oxygen saturation (SmO_2_) and improve maximal grip strength and pressure pain threshold compared with distal-to-proximal and placebo taping.

To achieve this, a randomized controlled trial design was implemented with four intervention groups: KT applied with proximal-to-distal tension, KT applied with distal-to-proximal tension, a third with no tension (placebo), and a control group without any taping. This approach seeks to provide rigorous and objective evidence to clarify the potential benefits of KT in sports.

Clinically, understanding whether kinesiotaping modulates local oxygenation, strength, or pain sensitivity has implications for injury prevention, recovery monitoring, and rehabilitation strategy optimization. The forearm musculature is often subjected to repetitive loading in sports and occupational activities, and while these conditions can lead to overuse syndromes such as lateral epicondylitis or tendinopathy, it is first necessary to characterize effects of Kinesio tape in healthy tissue. in healthy tissue. Studying healthy, physically active individuals allows researchers to isolate the direct mechanical and sensory influences of kinesiotaping without the confounding impact of pain, inflammation, or pathological adaptation. Establishing this physiological baseline is a critical first step in translating the mechanisms of KT into clinical and rehabilitation contexts. By exploring whether taping direction can modify objective parameters such as muscle oxygen saturation (SmO_2_), pressure pain threshold (PPT), and grip strength, this study provides foundational evidence that may guide future applications in injured or fatigued populations.

## 2. Materials and Methods

This study was conducted in accordance with the guidelines of the Declaration of Helsinki and was approved by the Research Commission of the European University’s Doctoral School and Research with authorization code 2025-155. This study was registered at ClinicalTrials.gov (registration number NCT06993857) to ensure methodological transparency and adherence to international standards for clinical research. All participants signed an informed consent form before participating, which detailed the potential risks and benefits of the intervention. All personal data and individual results will be treated with strict confidentiality.

### 2.1. Study Design

This study followed the CONSORT guidelines for experimental studies (Supplementary Materials Table S1) [13]. A controlled experimental design was chosen with a randomized allocation of forearms to four intervention conditions. Pre- and post-intervention measurements were performed in a single session. This structure allowed for the analysis of the immediate effects of different KT application methods (proximal–distal tension, distal–proximal tension, no tension, and no tape) on physiological and neuromuscular variables in highly controlled conditions.

All participants were right-handed, as determined by self-report, to minimize the variability related to limb dominance. Both forearms were evaluated under identical testing conditions, and random assignment ensured a balanced distribution of the taping conditions between the dominant and non-dominant limbs. Each forearm was considered an independent experimental unit and was randomly allocated to one of four intervention conditions (proximal-to-distal, distal-to-proximal, placebo, or control).

The choice of this design was based on the objective of exploring acute changes and physiological mechanisms of action, while minimizing the influence of confounding factors related to adherence, inter-week variability, or the environment. The use of both forearms as independent units per subject maximized the sampling efficiency without compromising the internal validity of the study, while ensuring a balanced allocation between groups.

### 2.2. Participants and Eligibility Criteria

Participants were selected based on a series of well-defined inclusion and exclusion criteria to ensure the reliability and internal validity of this study. The inclusion criteria required individuals to be between 18 and 30 years of age, of any gender, and to maintain a physically active lifestyle. Specifically, participants had to engage in at least 150–300 min of moderate-intensity physical activity or 75–150 min of vigorous-intensity activity per week, sustained over a minimum period of six months. All participants were assessed in a resting state to avoid the influence of fatigue on the outcomes.

To be eligible, individuals had to present intact skin on the forearm and be free from any musculoskeletal disorders in the three months prior to this study. Additionally, participants could not have any diagnosed neurological or circulatory diseases, nor could they be undergoing any concurrent exercise programs or therapeutic interventions during the study period.

Conversely, the exclusion criteria were designed to eliminate the potential confounding variables. Individuals were excluded if they had suffered an acute soft tissue injury in the upper limb within the last month, had a history of surgical procedures affecting the upper limbs, or presented with functional impairments related to vision or vestibular sensitivity. Participants with known allergies to kinesiology tape (KT) or similar products, as well as those with frequent prior use of KT on the upper limbs, were also excluded to avoid desensitization effects. Furthermore, anyone who had received analgesic treatment for musculoskeletal pain in the three weeks preceding the study or had participated in a physical therapy rehabilitation program within the last three months was not considered eligible for the study. Finally, individuals with serious physical injuries that could interfere with the intervention were excluded.

### 2.3. Subject Recruitment and Randomization

Participants were recruited through an online campaign coordinated by our research team. All participants were active students at the Universidad Europea de Madrid (Villaviciosa de Odón, Spain). The experimental procedures were carried out in the Research Laboratories of the Universidad Europea de Madrid under controlled temperature and lighting conditions.

All kinesiotape applications were performed by a licensed physiotherapist with more than five years of clinical experience and a formal certification in kinesiotaping techniques, ensuring methodological consistency and proper tension application in all participants.

Randomization was conducted using a computer-generated random sequence created by an independent researcher who was not involved in the data collection or analysis stages (Random.org). Allocation concealment was maintained using sealed opaque envelopes that were opened only by the physiotherapist responsible for the tape application.

A total of 56 forearms from 28 participants were included in this study. A simple randomization procedure was applied separately for each forearm to ensure a balanced group distribution and minimize selection bias. Four intervention conditions were used (proximal-to-distal, distal-to-proximal, placebo, and control), resulting in 14 forearms in each group.

The researchers performing the post-intervention assessments and the statistician conducting the data analysis were blinded to the group allocation throughout this study. No changes were made to the methods after the trial had commenced.

### 2.4. Intervention Groups

**Figure 1:**
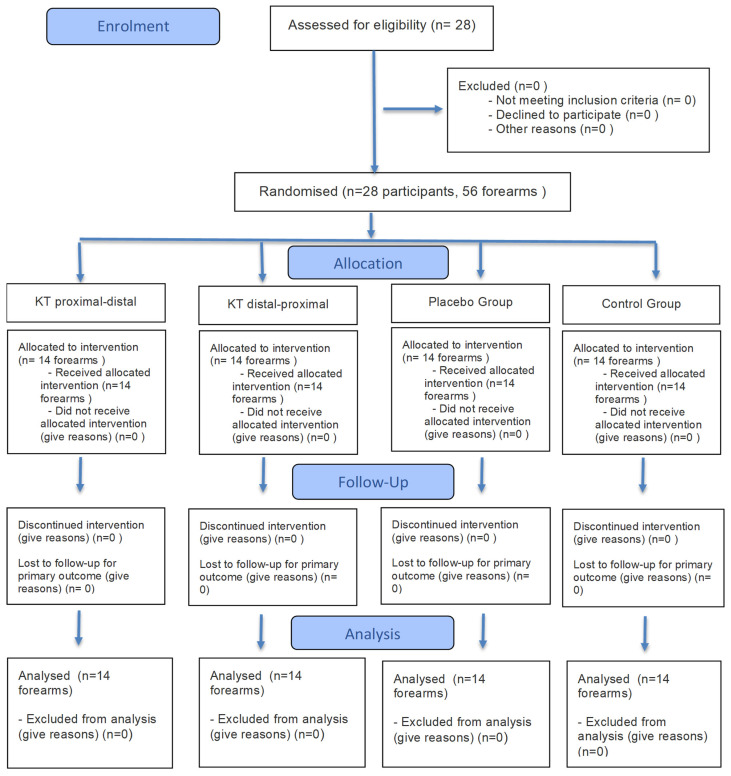
CONSORT flow diagram.

Participants were randomly assigned to one of four intervention groups: Group 1 received KT applied to the forearm with proximal-to-distal tension pattern. Group 2 received KT with a distal-to-proximal tension. Group 3 served as the placebo group, in which KT was applied without tension. Group 4 served as the control group and did not receive any KT application (Figure 1).

For the active kinesiotaping condition, the tape (Kinesio Tex Gold™, Kinesio Holding Corp., Albuquerque, NM, USA) was applied with approximately 50% of its maximum elastic tension, as recommended by the manufacturer. In the proximal-to-distal group, the tape was applied from the lateral epicondyle to the wrist, whereas in the distal-to-proximal group tape was applied in the reverse direction. The placebo group received the same tape applied without tension (0% stretch), and the control group did not receive any taping. The total tape length was standardized relative to each participant’s forearm length to ensure comparable application across individuals.

### 2.5. Sample Size and Statistical Power

The original sample size justification was based on a one-way analysis of variance (ANOVA) in G*Power (version 3.1). We specified a medium effect size (partial eta squared η^2^p = 0.14; equivalent Cohen’s f ≈ 0.40), two-sided alpha of 0.05, and 80% power. This analysis indicated that a minimum of 14 experimental units per group would be required to detect statistically significant differences with sufficient power across the four intervention arms. Considering that each participant could contribute both forearms as experimental units, we planned to recruit 28 subjects (totaling 56 forearms), enabling an equal allocation of 14 forearms per group. The choice of a medium target effect is supported by prior literature showing clinically relevant changes in handgrip strength with kinesiology taping in healthy individuals (Lemos et al., 2015) [14].

Given the bilateral design, observations were clustered within participants (two forearms per subject), and the nominal number of forearms overstated the independent information. Therefore, we accounted for within-subject correlation using the standard design effect DE = 1 + (m − 1) × ICC, with m = 2 (Donner and Klar, 2000) [15]. Under plausible intraclass correlations (0.05–0.30), the effective per-group sample size for the four-arm ANOVA was reduced from 14 to approximately 13.33–10.77 units. This implies a 5–30% loss of effective information relative to the independence assumption and, accordingly, some attenuation of the nominal 80% power unless the ICC is near zero.

To align the sample size logic with the analysis, we prespecified that inferential models would treat the forearms as repeated clustered observations within participants. Primary hypothesis testing was planned using linear mixed models with a random intercept for the subject (exchangeable correlation) or, alternatively, generalized estimating equations with an exchangeable working correlation, both adjusting for baseline values. From a sensitivity perspective focused on the primary contrast of substantive interest (pooling the two active KT arms versus placebo plus control), the nominal allocation of 28 versus 28 forearms corresponds—after clustering adjustment—to an effective per group size of approximately 26.67–21.54 across ICC = 0.05–0.30. This framing reflects the precision achieved under clustering and justifies the use of models that explicitly account for within-subject correlations.

### 2.6. Outcomes and Measurement Instruments

Maximal grip strength was defined as the primary outcome variable, reflecting functional forearm capacity and serving as the main indicator of the effectiveness of kinesiotaping. Muscle oxygen saturation (SmO_2_) and pressure pain threshold (PPT) were defined as secondary outcome variables, included to provide complementary information on local physiological and sensory responses.

Grip strength was assessed using a digital hand dynamometer (EH106/108, Shenzehen, China). The device was factory-calibrated before to data collection, following the manufacturer’s quality control protocol. Previous studies using Medi-Ción and comparable digital dynamometers have reported excellent test–retest reliability for maximal grip strength assessment in healthy adults (intraclass correlation coefficient [ICC] = 0.95–0.99) [16].

Muscle oxygen saturation (SmO_2_) and total hemoglobin (THb) levels were recorded using a portable near-infrared spectroscopy device (Moxy Monitor, Fortiori Design LLC, Spicer, MN, USA). The NIRS device was factory-calibrated before testing and has shown high reproducibility for assessing tissue oxygenation during exercise protocols (coefficient of variation < 5%) [17].

The pressure pain threshold (PPT) was measured using a digital algometer (Wagner Instruments FPX, Greenwich, CT, USA). This instrument has demonstrated excellent intra- and inter-rater reliability for musculoskeletal assessment (ICC = 0.90–0.98) [18].

All experimental procedures were conducted in the Research Laboratories of Universidad Europea de Madrid (Spain) under controlled temperature and lighting conditions. All testing sessions were conducted in the morning (between r and 12:00 a.m.) to minimize the potential influence of circadian variations on physiological and performance measures.

### 2.7. Intervention Protocol

This study followed a structured protocol comprising an initial evaluation (T1), a targeted exercise regimen designed to induce forearm muscle fatigue, and a final postexercise assessment (T2).

#### 2.7.1. Initial Evaluation

Upon arrival, the participants provided demographic data, including age, sex, weight, height, and body mass index (BMI). After signing the informed consent form, the participants were randomly assigned to one of the four intervention groups, as previously described. Forearm length was measured in centimeters using a measuring tape, with the participant seated in a standardized position. This value was used to determine the appropriate KT length according to the group allocation. Muscle oxygenation (SmO_2_) and total hemoglobin (THb) were assessed at rest using a Moxy Monitor placed over the flexor carpi radialis, with readings taken at the two-minute mark. Grip strength was measured using a digital dynamometer through three maximal efforts per hand.

**Figure 2:**
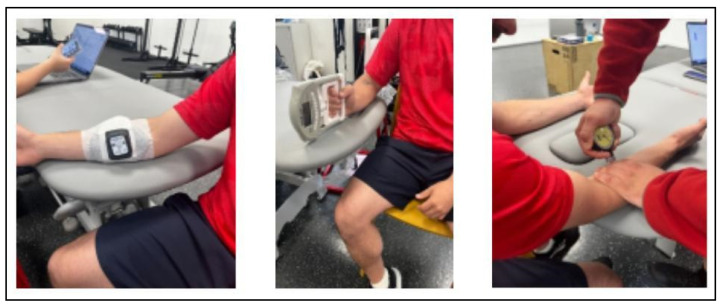
Measurement procedures for muscle oxygenation, total hemoglobin, grip strength, and pressure pain threshold.

The pressure pain threshold (PPT) was measured using a digital algometer at a standardized point over the flexor-pronator muscle group, approximately 2 cm distal and medial to the medial epicondyle. This location corresponds to the common flexor tendon origin, an area frequently affected by medial elbow overuse syndromes such as medial epicondylalgia (Figure 2).

**Figure 3:**
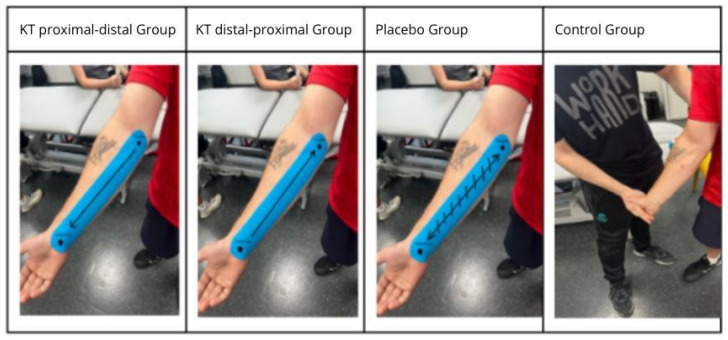
KT application by study group.

KT was applied by a certified physiotherapist following the standardized procedures. The forearm skin was cleaned with alcohol prior to the application. The participants were seated with their elbows fully extended, forearms in full supination, and wrists in full extension. The tape was placed along the flexor carpi radialis muscle from the medial epicondyle to the base of the second metacarpal. Tension was standardized by measuring the forearm length and subtracting 25% from the central portion of the tape, leaving 4 cm untensioned at each end (Figure 3).

#### 2.7.2. Exercise Protocol

The fatigue protocol was designed to induce acute muscular fatigue in the forearm flexor compartment under controlled conditions. The participants performed a standardized sequence of resistance and grip-based exercises targeting the wrist and finger flexors, extensors, radial and ulnar deviators, and pronator-supinator muscles. The protocol was executed sequentially for each arm, beginning with the dominant side to ensure a consistent effort distribution.

Each exercise was performed for three sets of 15 repetitions at a moderate-to-high intensity, corresponding to 60–70% of the individual’s maximal voluntary effort, as determined during the familiarization trials. The Rate of Perceived Exertion (RPE) was monitored using the Borg 0–10 scale, with the participants maintaining an effort level of ≥7/10 throughout the protocol.

Exercises were performed using a wrist roller bar (2.5 kg load), elastic resistance bands, and handgrip squeezes at a cadence of approximately one repetition per second. Minimal rest intervals (<30 s) were allowed between sets to maintain metabolic stress and ensure continuous loading of the forearm musculature. The between-arm transition periods did not exceed 2 min.

The total duration of the fatigue protocol was approximately 12–15 min per participant, depending on individual tolerance. Fatigue was confirmed operationally by a ≥20% reduction in maximal grip strength relative to baseline, consistent with prior fatigue-induction methodologies in upper-limb performance studies [19].

All exercises were supervised by the same physiotherapist to ensure correct execution and adherence to the prescribed intensities. Immediately after completing the final set, participants proceeded to the post-fatigue evaluation phase (Section 2.7.3) after removing the kinesiotape.

A detailed description of the specific exercises, target muscles, repetitions, rest periods, and load progression is provided in Supplementary Materials to facilitate the full reproducibility of the protocol.

#### 2.7.3. Post-Exercise Evaluation

The KT was removed immediately after completing the fatigue protocol. The second round of measurements was conducted in the following order: muscle oxygenation, grip strength, and pressure pain threshold. All assessments were performed using the same procedures as those used in the initial evaluation. Data were recorded directly in a digital database to ensure accuracy and traceability.

#### 2.7.4. Session Timeline and Duration

All experimental procedures were completed in a single testing session lasting approximately 60 min per participant. The standardized timeline was as follows:

- Initial evaluation and anthropometric measurements: ~10 min.
- Baseline (pre-intervention) assessments: 10 min, including measurements of muscle oxygenation (SmO_2_ and THb), maximal and average grip strength, and pressure pain threshold (PPT). These measurements were conducted without kinesiotape to establish reference values.
- Kinesiotape application: approximately 10 min per forearm, performed by a certified physiotherapist according to group allocation.
- Fatigue protocol: 10–15 min, conducted with kinesiotape in place for the KT and placebo groups, and without tape for the control group.
- Post-fatigue evaluation: Immediately after completing the fatigue protocol, the kinesiotape was removed and the same measurements (SmO_2_, THb, grip strength, and PPT) were repeated without the kinesiotape. This phase took approximately 10 min.
- Data verification and participant debriefing: 5 min.

### 2.8. Data Analysis

All statistical analyses were performed using SPSS software (version 29.0; IBM Corp., Armonk, NY, USA). The significance level was set at *p* < 0.05 for all tests. Prior to inferential analyses, the normality of continuous variables was verified using the Shapiro–Wilk test, and the homogeneity of variances was assessed using Levene’s test. All quantitative data met these assumptions and are expressed as the mean ± standard deviation.

Baseline differences among groups were evaluated using one-way analysis of variance (ANOVA) for continuous variables and chi-square tests for categorical variables. Although linear mixed models were initially considered to account for potential within-subject correlations (two forearms per participant), preliminary inspection indicated minimal clustering effects (ICC < 0.10). Therefore, a two-way repeated-measures ANOVA (time × group) was used as the primary inferential approach to assess the intervention effects.

When significant main or interaction effects were observed, Bonferroni-adjusted post hoc pairwise comparisons were conducted to explore simple effects. Within-group comparisons were used to evaluate pre–post changes for each intervention, while between-group comparisons were used to examinate post-intervention differences across groups. Effect sizes were calculated using partial eta squared (η^2^p) for ANOVA and Cohen’s d for pairwise contrasts, interpreted as small (<0.06/0.20), moderate (0.06–0.13/0.50), or large (≥0.14/0.80).

### 2.9. Data Management and Quality Control

All data were independently entered by two researchers into a secure database. Automatic range and consistency checks were performed prior to the analysis to detect potential data entry errors or outliers. Any discrepancies were resolved by cross-verifying the original source records to ensure the integrity of the data.

## 3. Results

### 3.1. Baseline Characteristics

A total of 28 physically active participants completed this study. The intervention was applied randomly to each participant’s forearm, which was then allocated to one of four intervention groups.

All quantitative demographic variables (age, weight, height, and BMI) met assumptions of normal distribution (Shapiro–Wilk test) and homogeneity of variances (Levene’s test) (*p* > 0.05 for all). One-way ANOVA revealed no statistically significant between-group differences for age (F [3,52] = 0.30, *p* = 0.820), weight (F [3,52] = 0.06, *p* = 0.982), height (F [3,52] = 1.01, *p* = 0.403), or BMI (F [3,52] = 0.09, *p* = 0.963).

These results confirm the effectiveness of the randomization procedure and the comparability of groups at baseline (Table 1).

### 3.2. Summary of Two-Way ANOVA Results

Table 2 presents the results of the two-way repeated-measures ANOVA (group × time). Significant main effects were found for SmO_2_ (time and interaction effects), and algometry (group effect). No significant interaction effects were identified for total hemoglobin (THb) or grip strength variables. Bonferroni-adjusted post hoc pairwise comparisons were conducted where significant effects were detected to explore within- and between-group differences.

### 3.3. Maximum Grip Strength

All groups showed significant reductions in maximal grip following the intervention, confirming the effectiveness of the fatigue protocol (Table 3). Within-group analysis revealed decreases in all conditions: KT distal–proximal (Δ = −7.92 kg, *p* = 0.001, Cohen’s d = −0.87), control (Δ = −5.12 kg, *p* = 0.001, Cohen’s d = −0.94), KT proximal–distal (Δ = −4.44 kg, *p* = 0.001, Cohen’s d = −0.80), and placebo (Δ = −4.68 kg, *p* = 0.001, Cohen’s d = −0.94). The two-way ANOVA revealed a significant main effect of time (F [1,52] = 18.73, *p* < 0.001, η^2^p = 0.14), but no significant group × time interaction (F [3,52] = 0.40, *p* = 0.752, η^2^p = 0.01). These results indicate that direction of Kinesiotape application did not differentially influence post-fatigue recovery of maximal grip strength.

### 3.4. Average Grip Strength

Average grip strength also decreased significantly across all groups after the fatigue protocol (Table 3). KT proximal–distal showed the greatest reduction (Δ = −8.05 kg, *p* = 0.001, Cohen’s d = −1.09), followed by KT distal–proximal (Δ = −5.16 kg, *p* = 0.011, Cohen’s d = −0.59). The two-way ANOVA revealed a main effect of time (F [1,52] = 14.95, *p* < 0.001, η^2^p = 0.13) but no significant interaction effect (F [3,52] = 0.80, *p* = 0.497, η^2^p = 0.02). These results confirm that although fatigue significantly impaired grip performance, kinesiotaping direction did not modulate this response.

### 3.5. Pressure Pain Threshold (Algometry)

As presented in Table 3, the control group exhibited the greatest reduction in pain sensitivity (Δ = −0.58 N, *p* = 0.021, Cohen’s d = −0.40), followed by KT proximal–distal (Δ = −0.47 N, *p* = 0.031, Cohen’s d = −0.30). The KT distal–proximal and placebo groups showed smaller, non-significant changes (*p* > 0.05). ANOVA results revealed a significant main effect of group (F [3,52] = 3.20, *p* = 0.027, η^2^p = 0.07), but no time or interaction effects (*p* > 0.23). These findings suggest minor group differences in pain threshold between groups, although not attributable to kinesiotaping direction.

### 3.6. Muscle Oxygen Saturation (SmO_2_)

All groups, including the control group, exhibited increases in SmO_2_ from pre- to post-intervention (Table 4). The KT proximal–distal group showed the largest improvement (Δ = +22.38, *p* = 0.001, Cohen’s d = 2.99), followed by the control group (Δ = +10.95, *p* = 0.042, Cohen’s d = 1.67). Although KT distal–proximal (*p* = 0.082) and placebo (*p* = 0.193) did not reach significance, both showed large effect sizes (Cohen’s d > 1.1). The ANOVA revealed significant time (F [1,52] = 94.51, *p* < 0.001, η^2^p = 0.48) and interaction effects (F [3,52] = 5.49, *p* < 0.001, η^2^p = 0.32). Post hoc Bonferroni comparisons confirmed that the KT proximal–distal group experienced a significantly greater increase in SmO_2_ than all other groups (*p* < 0.05). These findings indicate that taping direction significantly influences muscle oxygenation, with proximal–distal application producing the pronounced facilitative effect.

### 3.7. Total Hemoglobin (THb)

Post-intervention *THb* values increased slightly in all groups but did not reach statistical significance (*p* > 0.15 in all cases; Table 4). The two-way ANOVA revealed no significant main or interaction effects (group: F [3,52] = 1.97, *p* = 0.123, η^2^p = 0.05; time: F [1,52] = 2.50, *p* = 0.117, η^2^p = 0.03; interaction: F [3,52] = 0.64, *p* = 0.593, η^2^p = 0.13). These findings suggest that kinesiotaping did not produce measurable changes in total hemoglobin levels following the fatiguing protocol.

## 4. Discussion

This study investigated the acute effects of kinesiotaping (KT) applied to the forearm flexor muscles on maximal grip strength, pressure pain threshold, and muscle oxygenation in healthy, physically active adults following a fatiguing exercise protocol.

The main finding was a significant increase in muscle oxygen saturation (SmO_2_), particularly in the proximal-to-distal KT application. This suggests that the direction of taping can transiently enhance local muscle perfusion. In contrast, no significant effects were observed for maximal or average grip strength, pressure pain threshold, or total hemoglobin (THb), indicating that KT did not alter neuromuscular performance or pain perception under acute fatigue conditions.

These findings highlight that while KT may influence local oxygenation dynamics, it does not appear to directly affect functional or sensory outcomes in healthy individuals.

Regarding muscle strength, the absence of significant between-group differences aligns with the current literature indicating limited or no acute ergogenic effects of KT. For instance, Limmer et al., 2020 [2] reported that KT applied to finger flexors in climbers did not improve grip strength or sports performance, consistent with our findings in physically active participants. By contrast, Ameer et al., 2022 observed increased grip strength in sedentary women immediately after KT application, though their study lacked a fatigue protocol and differed in population and taping placement, limiting comparability [10]. Similarly, Li et al., 2024 found that KT enhanced post-fatigue strength in the ankle musculature of male students, suggesting that any performance-related effects may depend on muscle group, taping technique, and physiological context rather than a universal mechanism of action [20].

With regard to pain sensitivity, the present study did not detect significant changes in pressure pain threshold, indicating that KT did not acutely influence deep pressure–induced pain perception in healthy participants. This is consistent with Banerjee et al., 2019, who reported that KT may modulate the perception of superficial mechanical stimuli but has inconsistent effects on deep nociceptive responses [4]. Similarly, Güvener et al., 2024 observed reduced pain intensity in patients with carpal tunnel syndrome following KT application, despite the absence of structural changes on ultrasound imaging [21]. These findings suggest that the analgesic effects of KT are likely context-dependent, possibly emerging in chronic or pathological conditions rather than in healthy individuals after a single, acute application.

Therefore, our findings support the view that KT’s effects on pain modulation may involve longer-term neurosensory or circulatory adaptations, rather than immediate mechanical or sensory changes.

The physiological effects of KT on circulatory dynamics has been explored previously with mixed results. For example, Banerjee et al., 2020 [3] used laser Doppler imaging to evaluate forearm cutaneous blood flow and found no significant differences between KT, rigid taping, and control conditions, suggesting a limited effect on superficial microcirculation at rest. Similarly, Carvalho et al., 2024 used near-infrared spectroscopy (NIRS) in patients with chronic venous insufficiency and found no significant changes in tissue perfusion after KT application to the calf indicating that the hemodynamic effects of KT may depend on physiological state and muscle group [12].

In contrast, our study offers novel evidence that the proximal-to-distal KT application significantly increases muscle oxygen saturation (SmO_2_) following a fatiguing exercise protocol. The two-way ANOVA revealed significant main effects of time and a group × time interaction, confirming that the proximal-to-distal application produced a greater oxygenation response compared with other taping directions and control conditions. Interestingly, total hemoglobin (THb) did not differ significantly across groups, suggesting that the observed increase in SmO_2_ reflects enhanced oxygen utilization efficiency rather than increased blood volume.

These results imply that KT direction may transiently modulate local muscular perfusion or venous return, particularly under fatigued conditions, offering a physiological rather than clinical interpretation of KT’s mechanism of action.

Moreover, this study is among the first to use NIRS to evaluate the acute effect of KT on deep muscle oxygenation in the forearm of healthy, physically active adults. This methodology offers objective insight into the microvascular response of muscle tissue and supports the potential of NIRS as a valuable tool for future research on recovery and fatigue management in sports and rehabilitation contexts.

In line with previous studies, a recent meta-analysis by Stocco et al., 2024 concluded that KT does not contribute to muscle strength gains in athletes with or without musculoskeletal injuries [22]. A systematic review by Reneker et al., 2018 also established that there is no convincing evidence to support the use of KT for improving sports performance, comparing skills such as ball handling, agility, and dynamic balance [23]. The results of the present study are consistent with these findings, as they did not demonstrate significant acute effects of KT on strength or pain. However, a relevant physiological effect on muscle oxygenation was evidenced, which could open new lines of research to better understand its mechanism of action and its real applicability in a sports context. The findings of this study reinforce the need for caution in interpreting KT applications, especially when used for sports performance enhancement in healthy individuals.

Despite the controlled and rigorous nature of this trial, several limitations must be acknowledged. First, as this was a single-session, immediate post-intervention evaluation, the observed effects cannot be extrapolated to sustained physiological changes over time. Second, while the 56 forearms were treated as independent experimental units, the fact that two forearms came from the same participant could introduce an uncontrolled statistical dependency if not accounted for through advanced statistical models, such as mixed-effects models. Furthermore, the study did not evaluate the participants’ subjective perception, which could have provided complementary information on the sensory experience of the kinesiotape. Finally, the sample size, although sufficient to detect moderate effects, may limit the generalizability of the results to clinical populations or different functional contexts.

The findings of this study provide valuable information for physiotherapists in both rehabilitation and sports medicine, highlighting that the benefits sought from this type of tool should be approached with caution. A key strength of this study is the inclusion of muscle oxygenation measurement using NIRS with a Moxy Monitor. A key strength of this study is the inclusion of muscle oxygenation measurement using NIRS with a Moxy Monitor. NIRS technology allows for continuous, non-invasive assessment of local muscle metabolism and microvascular function, providing valuable insight into tissue oxygen extraction and perfusion dynamics. In healthy, physically active populations, NIRS has been widely used to monitor fatigue development, recovery status, and exercise efficiency [24]. In contrast, in clinical populations with circulatory or musculoskeletal disorders, such as peripheral arterial disease, chronic venous insufficiency, or tendinopathies, NIRS enables the detection of impaired oxygen delivery and utilization, offering potential applications for rehabilitation monitoring and treatment evaluation [25]. Therefore, integrating NIRS into KT research provides both physiological and translational relevance, bridging experimental findings with potential clinical implications.

Future research should expand on these findings by increasing sample size, including symptomatic or clinical populations, and assessing KT application during real-world dynamic sports scenarios. The use of NIRS represents a promising avenue for deepening our understanding of how different therapeutic interventions influence local muscle perfusion and recovery.

## 5. Limitations

One limitation of the present study is that subcutaneous fat thickness was not measured, which may influence NIRS signal quality and the accuracy of muscle oxygenation readings. However, as all participants were healthy and physically active with normal BMI values, variability due to adipose tissue thickness was likely minimal. Future studies should consider direct assessment of this parameter to improve the reliability and external validity of oxygenation measurements.

Despite careful methodological control, several potential sources of bias must be acknowledged. The exclusive inclusion of young, physically active university students may have introduced selection bias, limiting the generalizability of findings to clinical populations, individuals with musculoskeletal pain, or those with varying levels of physical fitness who may respond differently to KT application.

Moreover, although all taping procedures were performed by the same experienced physiotherapist following a standardized protocol, minor variations in tape tension, placement, or adhesion—possibly related to individual skin characteristics—may have affected local mechanical or physiological responses. Such variations are inherent to manual taping techniques and may contribute to inter-individual variability in outcomes.

Participants were not blinded to the presence or absence of tape, potentially introducing performance or expectation bias. However, to mitigate this, both assessors and data analysts were blinded to group allocation. Furthermore, since both forearms from the same participant were used as experimental units, there is a potential for within-subject dependency. While this was statistically addressed in the analysis, future research could benefit from more advanced statistical approaches (e.g., linear mixed models) to account for nested data structures.

Acknowledging these limitations enhances the transparency and internal validity of this study’s findings and provides a clearer context for interpretation.

## 6. Physiological Relevance and Potential Applications

The current findings offer valuable physiological insight into how KT direction may acutely influence local muscle oxygenation and function in healthy adults. The observed increase in muscle oxygen saturation (SmO_2_) following proximal-to-distal KT application suggests a transient enhancement in local oxygen delivery or reperfusion after fatigue, rather than a direct facilitation of blood perfusion.

In contrast, the lack of significant effects on maximal grip strength and pressure pain threshold supports the notion that KT does not acutely enhance neuromuscular performance or pain sensitivity in healthy tissue. As such, KT’s role may lie more in recovery monitoring and experimental physiology rather than as a performance-enhancing or analgesic intervention.

Although this study focused on healthy participants, the SmO_2_ improvement observed following proximal-to-distal taping could provide a foundation for future translational research. This might include early rehabilitation scenarios in conditions such as tendinopathy, where local perfusion deficits are known to delay tissue repair.

From a practical standpoint, the proximal-to-distal application may be relevant in recovery-oriented or endurance-based training sessions, where optimizing local oxygenation dynamics is a priority. Furthermore, the use of near-infrared spectroscopy (NIRS) proved effective as a monitoring tool, providing objective feedback on muscle oxygenation responses to both exercise and taping interventions.

Overall, these findings contribute experimental evidence on the acute physiological effects of KT on muscle oxygenation and function. However, they should be interpreted with caution given the short-term nature of the intervention and the study’s young, healthy sample. Future research should explore whether these findings can be generalized to clinical populations and whether repeated or prolonged application of KT might produce cumulative effects.

From a clinical translation standpoint, limitations include restricted external validity to populations with tendinopathy or chronic pain, and the transient duration of the observed effects. Longitudinal studies are warranted to determine whether repeated KT application can lead to measurable changes in perfusion, recovery rate, or symptom perception during rehabilitation programs (Table 5).

## 7. Conclusions

This randomized controlled trial demonstrated that, in healthy, physically active adults, kinesiotaping applied to the forearm did not produce significant acute effects on maximal grip strength, pressure pain threshold, or total hemoglobin concentration following a fatiguing exercise protocol.

Although the proximal-to-distal taping direction was associated with a statistically significant increase in muscle oxygen saturation (SmO_2_), a similar post-fatigue increase was also observed in the control group. This pattern suggests that the observed improvement in SmO_2_ may reflect a non-specific reperfusion or transient recovery response, rather than a direct effect of KT on perfusion.

These findings provide preliminary physiological evidence that KT may accompany or facilitate short-term post-fatigue recovery of local muscle oxygenation. However, they do not support its use as an ergogenic or analgesic intervention in healthy individuals. Therefore, the effects observed should be interpreted as physiological rather than clinical.

Future studies should determine whether similar SmO_2_ responses occur in clinical or athletic populations and whether repeated or long-term KT application could elicit cumulative adaptations in tissue perfusion, recovery kinetics, or fatigue resistance.

In summary, proximal-to-distal kinesiotaping appears to transiently enhance local muscle oxygenation following fatigue. While this represents a physiologically interesting observation, its clinical or performance relevance remains limited and requires further investigation.

## Figures and Tables

**Table 1:** Participant demographic data. Mean ± standard deviation.

Outcome	KT Proximal–Distal Group (n = 14)	KT Distal–Proximal Group (n = 14)	Placebo Group (n = 14)	Control Group (n = 14)	F [3,52]	*p*
Age (years)	23.51 ± 3.70	23.85 ± 3.58	24.55 ± 1.88	24.26 ± 2.78	0.30	0.820
Weight (kg)	68.88 ± 15.18	70.67 ± 14.08	69.92 ± 14.02	70.69 ± 9.98	0.06	0.982
Height (m)	1.72 ± 0.08	1.73 ± 0.07	1.69 ± 0.09	1.69 ± 0.08	1.01	0.403
BMI (kg/m^2^)	23.52 ± 2.77	23.50 ± 3.20	23.83 ± 2.82	23.28 ± 2.11	0.09	0.963

**Table 2:** Summary of Two-Way ANOVA Results (Group × Time) for All Outcome Variables.

Variable	Effect	F	*p*	η^2^p
SmO_2_	Group	2, 31	0.081	0.06
SmO_2_	Time	94, 51	<0.001	0.48
SmO_2_	Group × Time	5, 49	<0.001	0.32
THb	Group	1, 97	0.123	0.05
THb	Time	2, 5	0.117	0.03
THb	Group × Time	0, 64	0.593	0.13
Algometry	Group	3, 2	0.027	0.07
Algometry	Time	1, 43	0.235	0.01
Algometry	Group × Time	0, 18	0.913	0
Dynamometry Max	Group	2, 68	0.051	0.07
Dynamometry Max	Time	18, 73	<0.001	0.14
Dynamometry Max	Group × Time	0, 4	0.752	0.01
Dynamometry Avg	Group	0, 74	0.533	0.02
Dynamometry Avg	Time	14, 95	<0.001	0.13
Dynamometry Avg	Group × Time	0, 8	0.497	0.02

Max = Maximum; Avg: Average.

**Table 3:** Summary of Pre- and Post-Intervention Outcomes for Algometry and Dynamometry Across Experimental groups.

Outcome	Group	N	Mean Pre	SD_Pre	Mean_Post	SD_Post	Mean_Diff	*p*_Value	Cohen’s_d
Algometry	KT proximal–distal group	14	4.98	1.62	4.51	1.52	−0.47	0.031	−0.30
Algometry	KT distal–proximal group	14	5.85	1.67	5.51	1.78	−0.34	0.072	−0.20
Algometry	Placebo group	14	4.57	1.17	4.55	1.61	−0.02	0.220	−0.02
Algometry	Control group	14	5.66	1.45	5.08	1.41	−0.58	0.021	−0.40
Dynamometry Max	KT proximal–distal group	14	34.82	6.15	30.38	4.95	−4.44	0.001	−0.80
Dynamometry Max	KT distal–proximal group	14	40.86	10.49	32.94	7.47	−7.92	0.001	−0.87
Dynamometry Max	Placebo group	14	38.70	5.62	34.02	4.18	−4.68	0.001	−0.94
Dynamometry Max	Control group	14	38.91	7.72	33.79	0.41	−5.12	0.001	−0.94
Dynamometry Avg	KT proximal–distal group	14	36.22	8.61	28.17	5.89	−8.05	0.001	−1.09
Dynamometry Avg	KT distal–proximal group	14	36.94	9.68	31.78	7.74	−5.16	0.011	−0.59
Dynamometry Avg	Placebo group	14	35.77	4.39	32.11	3.93	−3.65	0.001	−0.88
Dynamometry Avg	Control group	14	36.05	6.35	33.23	4.72	−2.82	0.001	−0.50

**Table 4:** Summary of Pre- and Post-Intervention Outcomes for SmO_2_, THb across experimental groups.

Outcome	Group	N	Mean Pre	SD_Pre	Mean_Post	SD_Post	Mean_Diff	*p*_Value	Cohen’s_d
SmO_2_	KT proximal–distal group	14	53.46	3.79	75.84	9.88	22.38	0.001	2.99
SmO_2_	KT distal–proximal group	14	56.85	7.03	67.01	6.45	10.16	0.082	1.51
SmO_2_	Placebo group	14	61.10	6.08	69.62	8.40	8.52	0.193	1.16
SmO_2_	ControlG	14	61.45	7.17	72.40	5.87	10.95	0.042	1.67
THb	KT proximal–distal group	14	12.53	0.37	12.58	0.27	0.05	0.151	0.16
THb	KT distal–proximal group	14	12.75	0.52	12.82	0.42	0.07	0.172	0.15
THb	Placebo group	14	12.46	0.41	12.76	0.28	0.30	0.902	0.85
THb	Control group	14	12.61	0.53	12.67	0.51	0.06	0.203	0.12

**Table 5:** Clinical translation of study findings.

Finding	Physiological Interpretation	Clinical Application	Implication for Practice
Increased SmO_2_ after proximal–distal KT	Enhanced local oxygen delivery and microvascular response post-fatigue	May support post-exercise recovery or fatigue management in repetitive forearm activities	Consider KT as a short-term circulatory adjunct in recovery or preventive taping routines
No change in grip strength	Lack of acute neuromuscular facilitation effect	Not suitable for immediate strength enhancement	Use KT alongside active rehabilitation or motor control exercises
No change in PPT	Minimal short-term analgesic or sensory modulation	Limited value for acute pain relief in healthy tissue	Combine KT with other therapeutic modalities for pain management

## Data Availability

The raw data supporting the conclusions of this article will be made available by the authors on request.

## Supplementary Materials

The following supporting information can be downloaded at https://www.mdpi.com/article/10.3390/healthcare13243211/s1, Table S1: Detailed Fatigue Exercise Protocol.

## Abbreviations

The following abbreviations are used in this manuscript:

KT	Kinesiotaping
NIRS	Near-infrared spectroscopy
SmO_2_	Muscle oxygen saturation
THb	Total Hemoglobin

## References

[1] Williams S., Whatman C., Hume P.A., Sheerin K. (2012). Kinesio taping in treatment and prevention of sports injuries: A meta-analysis of the evidence for its effectiveness. Sports Med..

[2] Limmer M., Buck S., de Marees M., Roth R. (2020). Acute effects of kinesio taping on muscular strength and endurance parameters of the finger flexors in sport climbing: A randomised, controlled crossover trial. Eur. J. Sport Sci..

[3] Banerjee G., Briggs M., Johnson M.I. (2020). The immediate effects of kinesiology taping on cutaneous blood flow in healthy humans under resting conditions: A randomised controlled repeated-measures laboratory study. PLoS ONE.

[4] Banerjee G., Briggs M., Johnson M.I. (2019). The effects of kinesiology taping on experimentally-induced thermal and mechanical pain in otherwise pain-free healthy humans: A randomised controlled repeated-measures laboratory study. PLoS ONE.

[5] Hebert-Losier K., Yin N.S., Beaven C.M., Tee C.C.L., Richards J. (2019). Physiological, kinematic, and electromyographic responses to kinesiology-type patella tape in elite cyclists. J. Electromyogr. Kinesiol..

[6] Karahan A.Y., Yildirim P., Kucuksarac S., Ordahan B., Turkoglu G., Soran N., Ozen K.E., Zinnuroglu M. (2017). Effect of Kinesio taping on elbow muscle strength in healthy individuals: A randomized trial1. J. Back Musculoskelet. Rehabil..

[7] Zhong Y., Zheng C., Zheng J., Xu S. (2020). Kinesio tape reduces pain in patients with lateral epicondylitis: A meta-analysis of randomized controlled trials. Int. J. Surg..

[8] Drapeza R.C., Navasca S.B., Dones V., Rimando C.R. (2022). The effects of taping on de Quervain’s disease: A systematic review and meta-analysis. J. Bodyw. Mov. Ther..

[9] Giray E., Karali-Bingul D., Akyuz G. (2019). The Effectiveness of Kinesiotaping, Sham Taping or Exercises Only in Lateral Epicondylitis Treatment: A Randomized Controlled Study. PM R.

[10] Ameer M., Abbad A., Subbarayalu A., Alsharari A., AlRuwaili R., AlFuhigi S., Hmdan N., Alshammari A., Alhuthayl G. (2023). Immediate and localized effect of Kinesio tape on the hand grip strength of sedentary female adults. J. Med. Life.

[11] Feldmann A., Schmitz R., Erlacher D. (2019). Near-infrared spectroscopy-derived muscle oxygen saturation on a 0% to 100% scale: Reliability and validity of the Moxy Monitor. J. Biomed. Opt..

[12] Carvalho M.L.V., Caceres V.M., Nascimento I.O., Costa H.S., Figueiredo P.H.S., Lima V.P., Monteiro D.P., Pereira D.A.G. (2024). Is Kinesio taping acutely effective for peripheral tissue perfusion in women with mild to moderate chronic venous insufficiency? A randomized controlled trial. J. Bodyw. Mov. Ther..

[13] Rennie D. (2001). CONSORT revised—Improving the reporting of randomized trials. JAMA.

[14] Lemos T.V., Pereira K.C., Protassio C.C., Lucas L.B., Matheus J.P. (2015). The effect of Kinesio Taping on handgrip strength. J. Phys. Ther. Sci..

[15] Donner A., Klar N. (2000). Design and Analysis of Cluster Randomization Trials in Health Research.

[16] Chou P.H., Lin C.J., Chou Y.L., Lou S.Z., Su F.C., Huang G.F. (2002). Elbow load with various forearm positions during one-handed pushup exercise. Int. J. Sports Med..

[17] Crum E.M., O’Connor W.J., Van Loo L., Valckx M., Stannard S.R. (2017). Validity and reliability of the Moxy oxygen monitor during incremental cycling exercise. Eur. J. Sport Sci..

[18] France C.R., Rhudy J.L., McGlone S. (2009). Using normalized EMG to define the nociceptive flexion reflex (NFR) threshold: Further evaluation of standardized NFR scoring criteria. Pain.

[19] Reuter S.E., Massy-Westropp N., Evans A.M. (2011). Reliability and validity of indices of hand-grip strength and endurance. Aust. Occup. Ther. J..

[20] Li Y., Xia Y., Zhang D., Fu S., Liu M., Pan X., Liu H. (2024). Immediate effect of kinesiology taping on muscle strength, static balance and proprioception after eccentric muscle fatigue on ankle: A randomized cross-over trial. BMC Musculoskelet. Disord..

[21] Guvener O., Dag F., Sahin G., Ozcakar L. (2024). Immediate effects of Kinesio taping in carpal tunnel syndrome: A randomized controlled double-blind ultrasonographic study. J. Hand Ther..

[22] Stocco M.R., Del Antonio A., Oliveira R.G., Parreiras S.O., Andraus R.A.C. (2024). Does kinesio tape alter muscle strength in athletes?—Systematic review and meta-analysis. J. Bodyw. Mov. Ther..

[23] Reneker J.C., Latham L., McGlawn R., Reneker M.R. (2018). Effectiveness of kinesiology tape on sports performance abilities in athletes: A systematic review. Phys. Ther. Sport..

[24] Grassi B., Quaresima V. (2016). Near-infrared spectroscopy and skeletal muscle oxidative function in vivo in health and disease: A review from an exercise physiology perspective. J. Biomed. Opt..

[25] Hamaoka T., McCully K.K., Niwayama M., Chance B. (2011). The use of muscle near-infrared spectroscopy in sport, health and medical sciences: Recent developments. Philos. Trans. Ser. A Math. Phys. Eng. Sci..

