# Supplementary material for: Clinical Implications of Kinesiotaping for Forearm Muscle Function: Acute Effects on Grip Strength, Pain Sensitivity, and Muscle Oxygenation in Healthy Active Adults: A Randomized Controlled Trial
Source: Healthcare (Basel). 2025 Dec 8;13(24):3211. doi: 10.3390/healthcare13243211 (PMC12733196; doi:10.3390/healthcare13243211)
Supplement: Supplementary file 1 [file healthcare-13-03211-s001.zip › healthcare-3988706-supplementary.pdf]

Supplementary Materials

Table S1. Detailed Fatigue Exercise Protocol

Exercise	Equipment	Target Muscles	Sets × Reps	Load/Intensity	Rest Interval	Description
1. Handgrip squeezes	Digital dynamometer	Finger and wrist flexors	3 × 15	~70% MVC	<30 s	Sustained squeezes with maximal effort pace
2. Wrist flexion/extension	Dumbbell (2.5 kg)	Wrist flexors/extensors	3 × 15	2.5 kg	<30 s	Seated position, full ROM, controlled tempo
3. Radial/ulnar deviation	Elastic band	Flexor/extensor carpi radialis & ulnaris	3 × 15	Moderate resistance	<30 s	Full deviation range, controlled tension
4. Pronation–supination	Wrist roller bar (2.5 kg)	Pronator teres, supinator	3 cycles	2.5 kg	<1 min	Alternating controlled rotation
5. Wrist roller elevation	Wrist roller bar (2.5 kg)	Flexor–pronator complex	3 cycles	2.5 kg	—	Raising/lowering the roller vertically
